# Medical Students in the United States

**Published:** 1859-03

**Authors:** 


Medical Students in the United States.—So far as we have been able to learn, the number of students attending the different colleges in this country during the session of 1858–59, is larger than it was last year. From a published catalogue, we see that 570 were in attendance at the Jefferson Medical College; but we are unable to state the size of the classes at the other schools in this city. From our exchanges, we find the following reports of the number of students at some of our medical institutions, which we believe are reliable:—University of Louisiana, 306; St. Louis Medical College, 135; University of New York, 350; College of Physicians and Surgeons, 180; New York Medical College, 107; Medical Department of Harvard University, 139; Shelby College, 53; New Orleans School of Medicine, 140.

